# Personalized mHealth Intervention (StepAdd) for Increasing Physical Activity in Japanese Patients With Type 2 Diabetes: Secondary Analysis of Social Cognitive Theory Measurements of a Single-Arm Pilot Study

**DOI:** 10.2196/60221

**Published:** 2025-03-28

**Authors:** Kayo Waki, Syunpei Enomoto, Toshimasa Yamauchi, Masaomi Nangaku, Kazuhiko Ohe

**Affiliations:** 1 Department of Biomedical Informatics, The University of Tokyo, 7-3-1 Hongo, Bunkyo-ku, Tokyo, 113-0033, Japan, 81 03-38122111; 2 Department of Diabetes and Metabolic Diseases, The University of Tokyo, Tokyo, Japan; 3 Department of Planning, Information and Management, University of Tokyo Hospital, Tokyo, Japan; 4 Division of Nephrology and Endocrinology, The University of Tokyo, Tokyo, Japan

**Keywords:** social cognitive theory, mHealth, mobile health, behavior change, diabetes, diabetic, type 2 diabetes, walk, step, exercise, physical activity, walking, digital therapeutics, secondary analysis, personalization, coping, self-efficacy

## Abstract

**Background:**

A 12-week pilot of the StepAdd mobile health (mHealth) behavior change intervention based on social cognitive theory (SCT) saw an 86.7% increase in mean daily step counts among patients with type 2 diabetes. Due to the lack of exploration of theoretical implications in mHealth intervention studies, there is a need to understand the mechanism underlying the behavioral change to inform the future design of digital therapeutics.

**Objective:**

This study aimed to examine the SCT drivers underlying the mean increase in exercise among Japanese patients with type 2 diabetes who participated in the StepAdd intervention.

**Methods:**

This is a post hoc analysis of data collected in the single-arm pilot study of the 32 patients who completed the StepAdd intervention. The StepAdd app uses self-mastery and coping strategies to increase self-efficacy and thus increase walking. Self-mastery was measured by the goal completion (GC) rate, which is the percentage of days in which patients met these adapting goals. The use of coping strategies was measured by the strategy implementation (SI) rate, which is the percentage of days in which patients applied their selected coping strategies. We assessed correlations between GC, SI, and self-efficacy to increase walking via linear regression and analyzed relationships via structural equation modeling.

**Results:**

We found statistically significant support for the SCT approach, including a correlation coefficient (ρ) of 0.649 between step increase and GC rate (*P*<.001); a ρ of 0.497 between the coping SI rate and self-efficacy increase (*P*=.004); a ρ of 0.446 between GC rate and self-mastery increase (*P*=.01); and a ρ of 0.355 between self-regulation increase and step increase (*P*=.046), giving us insight into why the behavior intervention succeeded. We also found significant correlations between self-efficacy for barriers and self-efficacy for task-specific behavior (ρ=0.358; *P*=.04), as well as self-regulation and self-efficacy for task-specific behavior (ρ=0.583; *P*<.001). However, a cross-lagged panel modeling analysis found no significant evidence that changes in self-efficacy preceded behavior changes in line with SCT.

**Conclusions:**

Self-mastery and coping strategies contributed to the walking behavior change in StepAdd, supporting the SCT model of behavior change. Future research is needed to better understand the causal pathways proposed by SCT.

## Introduction

Digital therapeutics (DTx), defined as evidence-based therapeutic interventions that are driven by high-quality software programs to treat, manage, or prevent a disease or disorder, can be effective in treating diabetes by inducing behavior changes [1]. However, studies of DTx generally do not explore the implications of the underlying behavior change theory. For example, we were unable to explain the mechanism behind behavior change with a randomized controlled trial (RCT) that showed significant improvement in hemoglobin A_1C_ (HbA_1c_) and albuminuria among patients with type 2 diabetes via DTx [2]. However, given the progress in DTx, it is not enough to simply show outcomes. Lifestyle change involves complex patient behavior comprising numerous decisions and significant effort. We need to explain the mechanism behind behavior change, to show the characteristics of the app in question that differ from those of other apps, and to explain the rationale for choosing the app to treat patients.

We analyzed results from a 12-week pilot study of 33 Japanese patients with diabetes who participated in the StepAdd mobile health (mHealth) behavior change intervention to explain the mechanism behind the behavior change [3]. This was a single-arm pilot study from August 2021 to December 2021 using pre-post evaluation to assess the feasibility and preliminary efficacy of a personalized mHealth intervention using the StepAdd app to improve physical activity among patients with type 2 diabetes, conducted in collaboration with Mitsui Memorial Hospital; Nihon Chouzai Co, Ltd (a community pharmacy); and Mitsui & Co, Ltd. Patients receiving type 2 diabetes treatment at Mitsui Memorial Hospital, with an HbA_1c_ of 7.5% or higher, were recruited by their physicians to participate in the pilot study evaluating the preliminary efficacy of StepAdd in increasing physical activity. StepAdd applies behavior change methods based on social cognitive theory (SCT) [4,5], and the pilot collected SCT-related measures throughout the intervention. The study included 6 visits spanning an initial step baseline phase and a 12-week continuous measurement phase. The transtheoretical model was used to categorize the participants who were currently at the contemplation stage (willing to change health behavior within the next 6 months), preparation stage (willing to change health behavior within the next month), or action stage (has made modifications to health behavior) to achieve the target goal of 10,000 steps a day. A community pharmacist briefed participants on this research study and on how to use the smartphone and the pedometer. The pilot saw an 86.7% increase in mean daily step counts, from 5436 (SD 2231) per day to 10,150 (SD 3908) per day, and a reduction of mean HbA_1c_ from 8.58% (SD 1.02%) to 7.79% (SD 1.11%) (−0.79%, SD 1.04%) [4]. A 24-week StepAdd RCT, with 80 participants per arm, is currently underway [6]. Analysis of RCTs has proven that increased walking causes clinically significant improvements to glycemic control among patients with diabetes [7,8]. The StepAdd pilot results show overall causality—the intervention caused the desired behavior and health improvement.

This secondary analysis seeks to use SCT measurements to understand the factors underlying the behavioral change, both to inform future analyses of the StepAdd RCT results and to improve the design of future interventions. We want to understand what features caused the change and whether the causal relationships defined by SCT can be proved.

SCT posits a central role to self-efficacy, the individual’s belief that they can perform the behavior, with increases in self-efficacy causing increases in the behavior. StepAdd uses self-mastery and coping strategies to increase self-efficacy and thus increase walking. For self-mastery, StepAdd implements achievable but challenging goals, which are adapted weekly, with self-mastery being measured by the goal completion (GC) rate. For coping strategies, StepAdd has participants choose a barrier and an associated coping strategy to implement, increasing self-efficacy, with coping strategy use being measured by the strategy implementation (SI) rate. During the StepAdd pilot, we collected 3 measures of the underlying latent construct of self-efficacy at weeks 0, 4, 8, and 12: self-efficacy in achieving the targeted behavior, self-efficacy in dealing with barriers, and self-regulation (SR).

## Methods

### Overview

This is a post hoc analysis of data collected in the StepAdd pilot for the 32 patients who completed the intervention (the pilot analyzed 33 patients, but 1 dropped out after week 4 because he expected the study to be just 4 weeks). Data came from pedometers, the StepAdd app, and questionnaire instruments, with data collected at weeks 0, 4, 8, and 12 of the intervention.

The step data came from daily counts from wireless pedometers (OMRON HJA-405 T-W or Yamasa AW-001), averaged for the 2 weeks prior to the week 0, 4, 8, and 12 time points.

The intervention used 2 SCT-based methods. First, it targeted improving self-mastery by using a series of attainable yet growing goals. The GC rate measured the percentage of days in which patients met these adapting goals [3]. Second, it targeted enhancing SR and improving sociocultural factors in the SCT framework by allowing patients to evaluate possible barriers to achieving their step goals and choose to implement associated coping strategies. The SI rate measured the percentage of days in which patients applied their selected coping strategies [3].

The intervention is based in particular on increasing self-efficacy, the belief in one’s own efficacy, applying it in this case to daily walking. We used 3 measures (self-efficacy for barriers [SE-B], self-efficacy for task-specific behavior [SE-T], and SR) of self-efficacy. SE-B is a self-efficacy scale to deal with barriers in achieving the targeted daily steps [9]. It is the sum of 4 questions, each rated 1‐5, delivered via a questionnaire, amounting to a total score range of 4‐20. SE-T is a self-efficacy scale for exercise in achieving the targeted daily steps [10]. It is the average of a participant’s confidence (in percentages) in their ability to walk 4000; 6000; 8000; 10,000; or 12,000 steps daily, as measured via a questionnaire. SR was measured using the Japanese version of the 12-item Physical Activity Self-Regulation Scale (PASR-12) [11]. It is the sum of 12 questions, each rated 1‐5, amounting to a total score range of 12‐60.

We analyzed measurements using means and standard deviations. We assessed correlation via linear regression and analyzed relationships via structural equation modeling. All statistical analyses used SAS 9.4, with the significance level for all the statistical tests at *P*<.05 (2-tailed). We calculated 95% CIs where applicable.

### Ethical Considerations

This study was approved by the Institutional Review Board of the University of Tokyo School of Medicine, under approval number 2021084NI-(2). This approval covers secondary analysis without additional consent. Study data were deidentified to ensure confidentiality and to protect all research participants.

## Results

**Figure 1.**
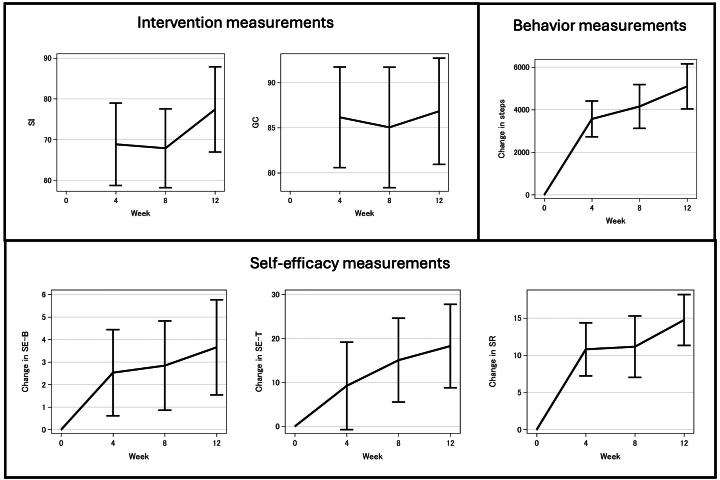
Means and 95% CIs of change in 6 measured items over 12 weeks in the StepAdd intervention for Japanese patients with type 2 diabetes. GC: goal completion; SE-B: self-efficacy for barriers; SE-T: self-efficacy for task-specific behavior; SI: strategy implementation; SR: self-regulation.

The behavior of interest, walking, increased steadily over the 12-week intervention (Figure 1). Both intervention method measurements (GC and SI) were consistently high (68%‐87%). The measures of self-efficacy (SE-B, SE-T, and SR) grew throughout the intervention.

**Figure 2.**
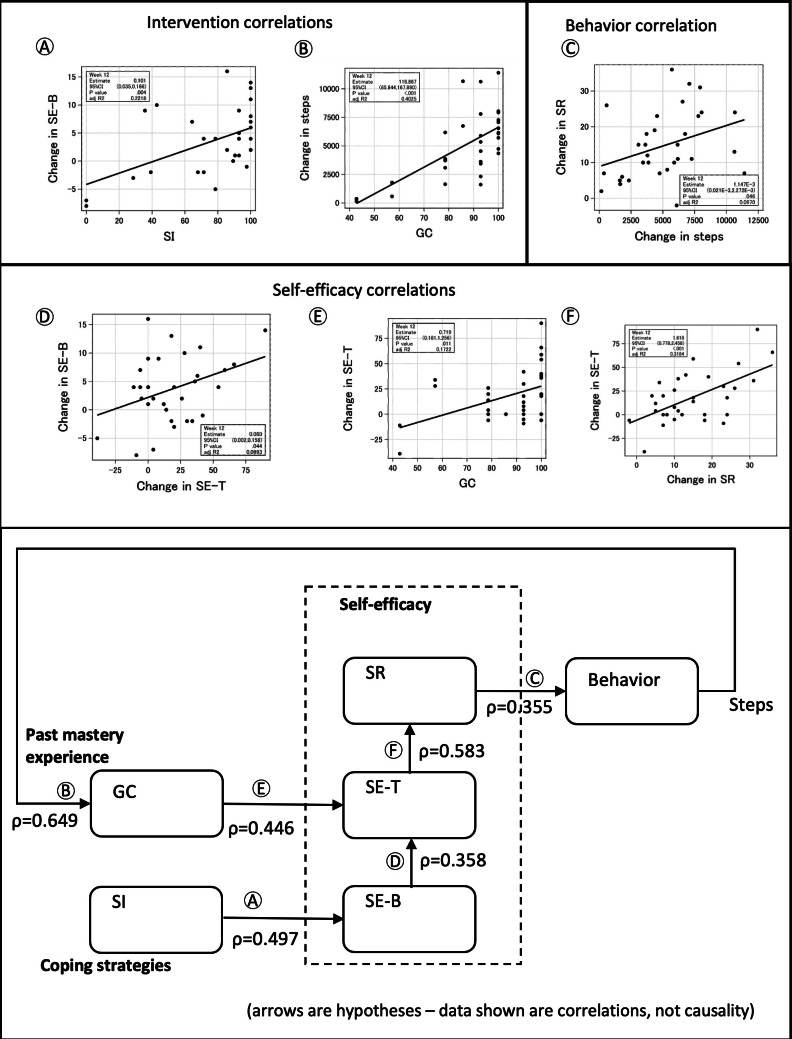
Social cognitive theory correlations at week 12 of the StepAdd intervention for Japanese patients with type 2 diabetes. (A) Intervention correlation between change in SE-B and SI. (B) Intervention correlation between change in steps and GC. (C) Behavior correlation between change in SR and change in steps. (D) Self-efficacy correlation between change in SE-B and change in SE-T. (E) Self-efficacy correlation between change in SE-T and GC. (F) Self-efficacy correlation between change in SE-T and change in SR. GC: goal completion; SE-B: self-efficacy for barriers; SE-T: self-efficacy for task-specific behavior; SI: strategy implementation; SR: self-regulation.

Analysis of the correlation of changes from baseline to week 12 among the 6 measurements revealed 6 relationships with statistical significance (Table 1 and Figure 2). When mapped to a simplified representation of SCT applied to this study, these relationships support but do not prove SCT’s central mechanism of changes in self-efficacy causing changes in behavior. The correlation coefficient (ρ) of 0.497 between SI and change in SE-B suggests that the coping strategies target of the intervention was effective. Similarly, the ρ of 0.649 between change in steps and GC and the ρ of 0.446 between GC and change in SE-T suggest that the self-mastery target of the intervention was effective. The ρ of 0.355 between change in SR and change in steps supports the centrality of SR in the changes to exercise behavior [3].

**Table 1.** Correlations of social cognitive theory constructs at week 12 of the StepAdd intervention for Japanese patients with type 2 diabetes.

	SI^a^	GC^b^	ΔSteps	ΔSE-B^c^	ΔSE-T^d^	ΔSR^e^
SI						
ρ	1					
*P* value	—^f^					
GC						
ρ	0.267	1				
*P* value	.140	—				
ΔSteps						
ρ	0.028	0.649	1			
*P* value	.879	<.001	—			
ΔSE-B						
ρ	0.497	0.254	0.134	1		
*P* value	.004	.161	.465	—		
ΔSE-T						
ρ	0.328	0.446	0.279	0.358	1	
*P* value	.067	.01	.122	.04	—	
ΔSR						
ρ	−0.073	0.297	0.355	0.348	0.583	1
*P* value	.691	.098	.046	.051	<.001	—

a SI: strategy implementation.

b GC: goal completion.

c SE-B: self-efficacy for barriers.

d SE-T: self-efficacy for task-specific behavior.

e SR: self-regulation.

f Not applicable.

We found significant correlations among changes in the 3 measures of self-efficacy (SE-T to SE-B and SR to SE-T). We used structural equation modeling to conduct a confirmatory factor analysis to explore how the 3 self-efficacy–related measures relate to a latent variable of self-efficacy. The analysis with 3 variables and the 32-participant dataset did not support an assessment of goodness-of-fit. The StepAdd RCT currently underway [6] targets 160 patients, and this larger dataset may support a more effective confirmatory factor analysis.

We used cross-lagged panel modeling to explore whether changes in the self-efficacy measures preceded changes in the behavior, a necessary condition for the causality postulated in SCT. The analysis with our 32 participants and 4-week data spacing did not reveal any significant cross-lagged correlations.

## Discussion

This study aimed to examine the SCT drivers underlying StepAdd’s mean increase in exercise. Overall, our analyses found statistically significant support for the SCT approach in the StepAdd intervention. Significant correlation was found for the following parameters: step increase and GC rate; SR increase and step increase; GC rate and self-mastery increase; and coping SI rate and self-efficacy increase.

Our study findings are consistent with a study that reported that interventions that focus on removing obstacles to intended planned fitness activities are effective in increasing self-efficacy to engage in exercise [12]. Effective use of coping strategies such as problem-solving has been shown to enhance self-efficacy, which in turn promotes engagement in physical activity [13]. Self-mastery, which has been shown to be one of the most important predictors of self-efficacy for physical activity in community-dwelling older adults, is also reflected in our study findings [14]. Achieving goals allows individuals to experience a sense of mastery with authentic evidence of their capabilities, which in turn boosts self-efficacy to increase physical activity [15]. Further, SR strategies such as self-monitoring and goal setting have been associated with exercise adherence in older adults [16].

Despite the significant correlations, there was a lack of support for directional associations from the cross-lagged panel modeling results. A key limitation of this study is the difference between the timescale of behavioral decisions and the measurement intervals. Participants made physical activity decisions multiple times per day, often in response to real-time feedback from the intervention. However, the study’s 4-week measurement intervals may not have fully captured these rapid behavior-feedback loops, making it difficult to assess causal relationships between intervention components and behavior change. Assessing causality is an area that requires further investigation. The pilot was relatively small, and further research is needed.

Our results provide insight into designing an effective DTx solution. Although some view DTx as centered on software implementation [17], we emphasize how DTx can remove time and space barriers, not just supporting patients but accompanying them 24/7 where they live, as a cost-effective low-labor augmentation to infrequent scheduled visits with health care professionals in a clinic.

Applying digital health to increase exercise levels is an active area of research [18], and more research is warranted. Interventions that are based on behavior change theory have been shown to be more effective than those not based on theory [19]. In our experience, building an intervention around a theory helps focus the effort on the key aspects, prioritizing them over less critical features. Our group has conducted numerous interventions not specifically based on a theory of human behavior change [2,20-23] that have produced results of interest, but we have found theory-based interventions [3,6,24] to be more effective. For example, the StepAdd study, which employs a theory-based approach, demonstrated a consistent increase in the average step count from week 1 to week 12 of the intervention [3]. In contrast, in the DialBetesPlus study, which is not theory-based, the average step count remained steady throughout the 12-month intervention period [2].

By understanding how the intervention relates to complex human behavior, researchers can put more emphasis on treatment elements that are effective over those that are not. Our results suggest that both of our intervention mechanisms, self-mastery and coping strategies, contributed to the change in behavior, supporting the SCT model of behavior change.

## Abbreviations

DTx: digital therapeutics
GC: goal completion
HbA_1c_: hemoglobin A_1c_
mHealth: mobile health
PASR-12: 12-item Physical Activity Self-Regulation Scale
RCT: randomized controlled trial
SCT: social cognitive theory
SE-B: self-efficacy for barriers
SE-T: self-efficacy for task-specific behavior
SI: strategy implementation
SR: self-regulation
